# KLF 6: a mitochondrial regulator in the kidney

**DOI:** 10.18632/oncotarget.4647

**Published:** 2015-06-25

**Authors:** Sandeep K. Mallipattu, John C. He

**Affiliations:** Department of Medicine/Nephrology, Mount Sinai School of Medicine, New York, NY, USA

Dysfunction of the glomerular filtration barrier is a major feature of Chronic Kidney Disease (CKD). Podocytes are terminally differentiated epithelial cells in the glomerulus that help maintain the integrity of the renal filtration barrier. Injury to the podocyte is the inciting event in glomerular diseases such as Minimal Change Disease (MCD), Focal Segmental Glomerulosclerosis (FSGS), and HIV-associated nephropathy (HIVAN). In many of these diseased conditions, podocyte injury contributes to a loss of differentiation markers, destabilization of the actin cytoskeleton, cell detachment, and eventual cell death. Although podocytes are terminally differentiated, they are dynamic in nature with high-energy requirements to maintain homeostasis [1]. Consequently, mitochondrial function is essential to meet these high-energy demands of the podocyte. Podocyte dysfunction as a result of mitochondrial injury has previously been reported in acquired and congenital glomerular disease [1]. However, transcriptional regulation of mitochondrial function in podocytes under stress is less defined. We previously reported that Krüppel-like factors (KLFs) play a critical role in regulating cell differentiation in the podocyte [2]. Initially discovered in the *Drosophila* in 1950, KLFs are a subfamily of DNA-binding zinc fingers involved in a diverse range of cellular processes [3]. In the past two decades, studies characterizing the essential role KLFs in maintaining homeostasis in epithelial and endothelial cells have dramatically risen.

By surveying the expression of *KLFs* in HIVAN cell culture model, we initially identified that *KLF6* expression was reduced in cultured human podocytes infected with HIV-1 [4]. Subsequently, we report that KLF6 is a critical mediator of mitochondrial function in the podocyte [4] by demonstrating the following: (1) KLF6 is an early inducible injury response gene, (2) podocyte-specific loss of *Klf6* increased the susceptibility to FSGS in mice, and (3) stable knockdown of *KLF6* in cultured human podocytes induced mitochondrial dysfunction and structural instability with eventual apoptosis under cell stress. In addition, restoration of KLF6 attenuated mitochondrial injury and prevented cell apoptosis. Interestingly, we also observe that podocyte-specific expression of KLF6 was significantly reduced in patients with FSGS as compared to healthy control subjects.

To the best of our knowledge, KLF6 is the first transcription factor critical to mitochondrial function under cell stress in the podocyte. Although, other transcription factors have been linked to podocyte apoptosis [5], their role in mitochondrial dysfunction has yet to be explored in the podocyte. Our studies suggest that KLF6 prevents mitochondrial injury by enhancing cytochrome c assembly via the regulation of cytochrome c assembly gene, *SCO2*. Several other genes involved in cytochrome c assembly were also reduced with the loss of *KLF6*, but it remains unclear whether the regulation of cytochrome c assembly is dependent solely on the KLF6-*SCO2* interaction. We postulate that KLF6 may complex with other factors to transcriptionally regulate several genes involved in enhancing cytochrome c assembly under cell stress. Further studies are required to explore these interactions to precisely decipher the mechanism by which KLF6 augments cytochrome c assembly.

Our studies with human podocytes demonstrate that restoration of KLF6 attenuates cell apoptosis [4]. However, KLF6 has diverse functions and the reinduction of KLF6 in the podocyte may result in activation of undesirable signaling pathways *in vivo*. Nonetheless, it is essential we determine whether the induction of KLF6 in mice with podocyte injury abrogates disease. Furthermore, targeting downstream targets of KLF6, such as *SCO2*, may prove to be a more specific therapeutic strategy in restoring cytochrome c assembly under cell stress.

The regulation of KLF6 under cell stress also needs to be further explored. Along with others, we observed that treatment with low-dose adriamycin induced the expression of KLF6 [4, 6]. However, it is unclear whether the regulation of cytochrome c by KLF6 is dependent on its response to DNA damage. For instance, KLF6 has been extensively studied in cancer [3] and the interaction between known inducers of DNA damage, such as p53 and p21, and KLF6 has also been explored [6]. In addition, Viale et al. demonstrated that surviving pancreatic cancer cells after oncogene ablation expressed a high dependence in mitochondrial respiration [7]. Consequently, a potential novel area of exploration in cancer biology is to examine the mechanism by which the regulation of cytochrome c assembly by KLF6 may contribute to the survival of cancer cells.
